# Dynamic knee joint function in children with juvenile idiopathic arthritis (JIA)

**DOI:** 10.1186/s12969-015-0004-1

**Published:** 2015-03-13

**Authors:** Sandra Hansmann, Susanne M Benseler, Jasmin B Kuemmerle-Deschner

**Affiliations:** Rheumatology, General Pediatrics, Oncology and Hematology University Children’s Hospital Tuebingen, Hoppe-Seyler-Str. 1, 72076 Tübingen, Germany; Rheumatology, Department of Pediatrics, Alberta Children’s Hospital, Calgary, Alberta Canada

**Keywords:** Juvenile idiopathic arthritis, Electrogoniometry, Joint function

## Abstract

**Background:**

Juvenile idiopathic arthritis (JIA) is a chronic illness with a high risk of developing long-term disability. Disease activity is currently being monitored and quantified by ACR core set. Here, joint inflammation is determined; however joint function is the crucial component for developing disability. The aim of this study was to quantify and compare dynamic joint function in healthy and arthritic knee joints and to evaluate response to improvement.

**Methods:**

A single center cohort study of consecutive children presenting to the rheumatology outpatient clinic was performed to measure dynamic knee joint function. Serial measures were performed if possible. Splint fixed electrogoniometers were used to measure dynamic knee joint function including ROM and flexion and extension torque.

**Results:**

A total of 54 children were tested including 44 with JIA, of whom eight had to be excluded for non-JIA-related knee problems. The study included 36 JIA patients of whom eight had strictly unilateral knee arthritis, and nine controls. Dynamic joint function ROM and torque depended on age and bodyweight, as demonstrated in healthy joints. ROM and torques were significant lower in arthritic compared to unaffected knee joints in children with unilateral arthritis and across the cohort. Importantly, extension torque was the most sensitive marker of impaired joint function. Follow up measurements detected responsiveness to change in disease activity.

**Conclusions:**

Measuring dynamic joint function with electrogoniometers is feasible and objective. Active ROM and torque during flexion and extension of arthritic knee joints were significant lower compared to unaffected. In dynamic joint measurement extension torque is a sensitive marker for disease activity.

## Key messages

- Dynamic knee joint function measurements using electrogoniometry are feasible in children with JIA
- Dynamic joint function was dependent on distinct factors including age and body weight.
- Extension torque measurements correlated well with clinical disease activity in children with JIA.

## Background

Juvenile Idiopathic Arthritis (JIA) is the most common chronic rheumatic disease in children with a prevalence of 1/1000. In many children, JIA is a life-long illness with a high risk of disease- and treatment-related morbidity [1,2]. Persistent inflammation can result in growth disturbances and deformities [3,4]. Children with JIA are commonly less active and have a lower quality of life [5-7]. Reduced physical activity results in decreased muscle strength [8], osteopenia and an increased fracture risk [9-12]. The fundamental therapeutic goal in JIA is achieving inactive joint disease in order to achieve and maintain normal joint function including range of motion (ROM), movement, coordination, and muscle strength and bone stability [13].

Precise assessments of joint function are crucial for treatment decisions in JIA [14]. In clinical practice, the joint status primarily captures the passive ROM of all joints plus frequently the child’s gait as a measure of functional impact. These assessments are subjective, results have limited inter-rater reliability. However, the pediatric ACR core set -the most widely used tool for treatment responsiveness in clinical routine and clinical trials – is centred around the physician-assessment derived number of active joints and joints with limitation of passive movement in addition to the physician global assessment, the patients/parents global assessment, the Child Health Assessment Questionnaire (CHAQ) and laboratory tests [15,16].

With increasing availability of highly effective JIA treatments, objective, sensitive and responsive instruments are urgently needed for monitoring therapies and predicting outcomes [17,18]. Hand-held-goniometry, hand-held-dynamometry and isokinetic measurements of muscle strength are tools to measure ROM, static grip strength and muscle strength in children with JIA [8,19]. Jumping mechanography and gait analyses are dynamic measurement tools to quantify a standardised jump or gait using force plates and camera systems [20-22]. Both methods were shown to be accurate, however expensive, time consuming and limited to a standardised, simulated setting, which is not on hand in most clinics.

Limited tools are available to capture joint function reliably and independent of the assessor. In daily practice, manual assessments of passive ROM lack accuracy and do not include the muscle function and torque [23]. For disease activity monitoring and outcome assessments other measurements of joint function are urgently needed. Electrogoniometry [24,25] is inexpensive, quick and easy to use. It may offer a feasible and assessor-independent strategy for the evaluation of the complete joint function with ROM, movement velocity, and flexion and extension torque.

The aims of the study were 1) to determine the dynamic knee joint function in a small group of healthy children using splint fixed electrogoniometry including the impact of age, gender, weight and handedness, 2) to determine the dynamic knee joint function in children with JIA and compare healthy and arthritic knee joints and 3) to analyze the impact of disease activity on dynamic knee joint function in JIA.

## Methods

### Patients

Consecutive patients presenting to the outpatient clinic of the Division of Pediatric Rheumatology at the University Children's Hospital Tuebingen for joint complaints over a period of 18 months were screened. Patients were eligible for the study, if they were 5 to 20 years of age and had a height ≤175 cm. All consenting patients underwent electrogoniometric measurements of the upper and/or lower extremity joints dependent on the location of their major complaint. During the study time period, 73 children had measurements of their dynamic joint function performed including 54 children with defined rheumatic illnesses, who had at least one knee joint measured, which constituted the Rheumatology Cohort.

Within this group, all children with JIA according to the Durban criteria [26] were identified and constituted the JIA cohort. Within this group an inception cohort was identified, including all children with evidence of acute unilateral knee arthritis, while the contralateral knee being currently and previously unaffected by arthritis or trauma. The control group consisted of age-matched children presenting to the outpatient clinic of the University Children's Hospital Tuebingen with two healthy knee joints and no history of arthritis, arthralgia, neuromuscular disorders or trauma.

Physical and musculoskeletal examination was performed by an independent pediatric rheumatologist not involved in this study or unrelated subsequent electrogoniometric measurements. All data were collected using the institutional electronic pediatric rheumatology documentation system ARDIS (Arthritis und Rheumatologie Dokumentation und Informationssystem). The study protocol was approved by the ethics committee of the Eberhard-Karls-University Tuebingen.

### Demographics, clinical and laboratory data

Demographic data included age, gender and handedness. Anthropometric measurements captured body weight and height plus circumference, length, height and width of the lower legs and feet. Disease-related information encompassed rheumatologic diagnosis including JIA and its subtypes, and other rheumatic diseases. In addition, JIA course, duration of illness and current and previous medications were noted. The joint status differentiated the categories of healthy joint, arthralgia with no evidence of arthritis, inactive arthritis, minimal residual arthritis and active arthritis. The number and sites of affected joints were noted, as well as the duration of arthritis. For each joint effusion, swelling, pain and limitation in range of motion (ROM) were recognized at routine clinical assessments. Leg length discrepancies were determined. Laboratory parameters included inflammatory markers (C-reactive protein, erythrocyte sedimentation rate), Antinuclear antibody, HLA B27 and rheumatoid factor. The Child Health Assessment Questionnaire (CHAQ) was completed at each visit.

### Dynamic knee joint function

Dynamic knee joint function was defined as active extent and strength of the knee joint movement into flexion and extension. The study captured range of motion (ROM), and dynamic muscle torque during flexion and extension.

### Measuring dynamic joint function

**Figure 1:**
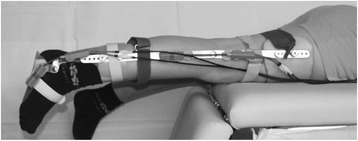
**Measuring dynamic joint function in juvenile idiopathic arthritis.** A 10-year old JIA patient wears two splint-fixed electrogoniometers. He is shown in neutral measurement position for active knee movements. The joint-angle over time is captured during active flexion and extension of the knee. Active ROM is defined as the maximum flexion and extension angle out of three cycles. Knee joint torque is calculated using the segment mass of the foot and lower leg and the acceleration at the start of flexion/extension movements.

Twin-axis electrogoniometers (Biometrics Ltd., Gwent, UK) were used to measure the joint angles during maximum flexion and extension movements. Each electrogoniometer consisted of a fixed and a telescopic end-block, linked with optical fibers to measure the variance of the joint angle over time during active joint movement. In the telescopic end-block mechanical signals were generated and converted into a digital signal by a data log unit. The joint angle was transferred and displayed on the linked monitor. The goniometers were splint-fixed to restrict knee movement to one single plain only [27]. Two different sizes of splints were available; each splint was adjustable to height and circumference of the participant’s lower extremity. The splints comprised of two parts, one fixed hard shell for the accommodation of the foot and lower leg and one cuff over the thigh. These two components were connected with an articulated splint which was changeable in length. The correct position of the splint parallel to the tibia was given due to the fixed hard shell around the lower leg and foot. In 90° flexion of the knee the correct length of the splint was positioned with the knee joint space direct under the articulation of the splint. The cuff over the thigh was fixed at the middle of the thigh and guaranteed that the splint was located parallel to the femur. The small splints were applied to children with a height less than 135 cm, the large splints to children with a height between 135 cm and 175 cm. The electrogoniometer was fixed with two stationary Velcro strips above the articulation of the splint, to guarantee the correct position during measurement. Participants were positioned prone with both knees extending the stretcher [28] (see Figure 1). This position facilitated the measurement of the range of motion (ROM) and the exact calculation of flexion and extension torque of the knee joint, since the thigh was fixed to the stretcher.

All participants were introduced to the dynamic joint function assessments using a standardized oral instruction describing the measuring procedure. Participants were asked to perform repeated full movement cycles in the maximum possible velocity with maximum extension and flexion. Measurements were performed consecutively of both knee joints. Three complete movement cycles were recorded. The measurement takes about 15–20 minutes including adjustment of splints, measurement and calculation of segment mass. All measurements were performed by two trained, experienced researchers. Pain was captured afterwards on a semi-quantitative scale. Patient satisfaction with the measurement procedure was documented. Serial measures of up to three times within a one-year period were obtained, if possible.

#### Dynamic Range of motion (ROM)

In clinical practice, single measurements of active and passive ROM represent the main components of the joint assessment in children with rheumatic illnesses. When using electrogoniometers, the joint-angle-over-time is captured in a standardized fashion during repeated dynamic measurements of joint flexion and extension at a sample rate of 250 Hz. The dynamic ROM reflects the maximum active joint angle over three flexion/extension cycles. It is therefore standardized and reproducible.

#### Flexion and extension torque

Segment mass is a prerequisite for the calculation of torque. Length and circumference of the lower leg as well as the length, width and height of the foot were measured at defined bony landmarks and used for the calculation of the volume and the associated mass of the extremity [29].

The movement of the knee is a rotational movement; the force applied to rotate the knee is the torque and is related to the dynamic maximum muscle force. During electrogoniometric measurements the flexion and extension torque was determined at the beginning of the movement utilizing the movement cycle with the maximum ROM. A low-pass filter type “Butterworth” with a cut-off-frequency of 6 Hz was used to eliminate artefacts. Acceleration was calculated during the movement from the measured angles. Flexion and extension torque during movement was then calculated using extremity mass and acceleration during movement taking into account the position in relation to gravitational force. The muscle torque was subsequently calculated electronically using the following formula.

$$ \begin{array}{c}\hfill {M}_K=\left[\left(\frac{1}{4}\cdot {m}_B\cdot \frac{1}{3}\cdot {m}_B\cdot {l}_B^2\right){\alpha}_K+\left[\frac{1}{12}\cdot {m}_F\cdot \left({d}_F^2+{l}_F^2\right)+{m}_F\cdot {\left(\frac{1}{2}\cdot {l}_F+{l}_B\right)}^2\right]\cdot {\alpha}_K\right]\hfill \\ {}\hfill -\left[\frac{1}{2}\cdot {l}_B\cdot {m}_B\cdot g\cdot \sin \left({\sigma}_K-{\varepsilon}_K\right)+\left(\frac{1}{2}\cdot {l}_F+{l}_B\right)\cdot {m}_F\cdot g\cdot \sin \left({\sigma}_K-{\varepsilon}_K\right)\right]\hfill \end{array} $$

The knee dynamic joint function is calculated using the formula above. Abbreviations: *M*_*K*_*:* muscle torque of the knee joint in Newton meter (Nm), *m*_*B*_: segment mass of lower leg in kg, *r*_*B*_: radius of the lower leg in meters, *l*_*B*_: length of the lower leg in meters, *m*_*F*_: segment mass of the foot in kg, *d*_*F*_: width of the foot in meters, *l*_*F*_: length of the foot in meters, *ε*_*K*_: measured angle during knee motion in rad, *σ*_*K*_: stationary angle in rad during knee motion of 90°, *α*_*K*_: angular acceleration during knee motion in rad/s^2^, *g*: acceleration of gravity in m/s^2^ (*g*=9.81 m/s^2^).

### Statistical analysis

Descriptive statistics were performed including frequencies, means and standard deviations, medians and ranges, as appropriate. Comparative analyses were conducted using parametric and non-parametric methods. Correlations of variables influencing the dynamic joint function were calculated using Pearson’s correlation coefficient, when appropriate. For intra-individual comparisons paired methods were applied.

## Results

### Patients

#### Rheumatology cohort

A total of 54 consecutive children had defined rheumatic diseases and were eligible for the study. These were 21 boys and 33 girls; median age at time of first measurement was 11.3 years. Mean disease duration was 20.9 months after diagnosis. All children had knee joint electrogoniometry performed (Table 1).

**Table 1:** **Baseline characteristics of children with juvenile idiopathic arthritis (JIA) and healthy controls tested for dynamic joint function**

	**Rheumatology cohort N = 54**
Gender, male: female	21:33
Median age in years (range)	11.3 (5.7 – 19.6)
Mean disease duration in month (range)	20.9 (0 – 161)
JIA subtypes	
• Oligoarticular arthritis	18
• Polyarticular arthritis	5
• Systemic arthritis	4
• Psoriatic arthritis	6
• Enthesitis-related arthritis	6
• Unclassified arthritis	5
Non-JIA rheumatic diseases	
• Dermatomyositis with arthritis	2
• Other	8
	**JIA cohort N = 36**
Gender, male: female	12: 24
Median age in years (range)	12.3 (5.7 – 19.6)
Mean disease duration in month (range)	25.8 (0 – 161)
JIA subtypes	
• Oligoarticular arthritis	
◦Extended	6
◦Persistent	9
• Polyarticular arthritis	4
• Systemic arthritis	3
• Psoriatic arthritis	5
• Enthesitis-related arthritis	5
• Unclassified arthritis	4
Joint status (66 knee joints tested)	
• active arthritis	14
• inactive arthritis, history of arthritis	28
• unaffected joints	24
Current treatments	
• methotrexate	10
• oral corticosteroidssteroids	5
• NSAIDs	15
• untreated	12
	**Inception cohort N = 8**
Gender male: female	2: 6
Median age in years (range)	10.5 (6.6 - 14.8)
Median height in cm (range)	141.8 (116.7 - 163.0)
Median weight in kg (range)	35.9 (20.7 - 57.1)
Handedness right/left/both	7/0/1
JIA disease duration after diagnosis	
≤1 month	4
>1 month	4
JIA subtypes	
• Oligoarticular arthritis	4
• Psoriatic arthritis	1
• Enthesitis-related arthritis	2
• Unclassified arthritis	1
Side of affected knee joint right: left	4: 4
Current treatments	
• NSAID	3
• untreated	5
	**Controls N = 9, 18 joints tested**
Gender male: female	5 4
Median age in years (range)	12.1 (7.3 – 19.3)
Median height in cm (range)	156.0 (130.0 - 168.1)
Median weight in kg (range)	42.9 (25.2 – 73.1)
Handedness right/left/both	7/1/1

*Abbreviation: NSAIDS* non-steroidal anti-inflammatory drugs.

#### JIA cohort

A total of 44/54 children (81%) had JIA, while 10 were diagnosed with other rheumatic diseases with associated arthritis. Eight JIA patients were excluded for additional, non-JIA related knee problems. Therefore, the total JIA cohort included 36 children, 12 boys and 24 girls with a median age of 12.3 years. The mean JIA disease duration was 25.8 months. A total of 66 joints were measured. Six patients only had one knee joint tested. Inception cohort: Within the JIA cohort, eight children had strictly unilateral knee arthritis, these were two boys and six girls; the median age was 10.5 years (Table 1).

The control group was small and consisted of only nine children. These were five boys and four girls, with a median age of 12.1 years. All anthropometric data of the controls are displayed in Table 1.

### Clinical data

In the JIA Cohort of 36 children the following JIA subtypes were present: oligoarticular arthritis in 15 children (42%) including 6 with extended oligoarthritis/polyarticular course, 4 (11%) with polyarticular arthritis, all rheumatoid factor negative, 3 (8%) with systemic arthritis, 5 (14%) with enthesitis-related arthritis (ERA) or psoriatic arthritis, respectively, and 4 (11%) with unclassified arthritis. A total of 14/66 (22%) knee joints had active arthritis, 28 (42%) joints were inactive after preceding arthritis and 24 (36%) were unaffected. Ten children were treated with methotrexate, oral steroids were given to five patients, 15 received NSAIDs and 12 were on no mediations at time of testing. Six patients (17%) received more than one medication at time of testing. All anthropometric data are summarized in Table 1.

The JIA patients of the inception cohort with strictly unilateral, active knee arthritis had oligoarticular arthritis in 50%, ERA in 25% and psoriatic or unclassified arthritis in 12.5%, respectively. Half of the children had right and half left knee arthritis. Half of the cohort were newly diagnosed with JIA and therefore had disease duration after diagnosis of ≤1 month. Fife of the eight children were measured prior to start of medication. Three children received NSAIDs. Anthropometric data are summarized in Table 1.

### Dynamic joint function in healthy children

The impact of anthropometric parameters including age, body weight, gender and handedness on dynamic joint function including ROM and flexion and extension torque was determined in healthy controls.

**Figure 2:**
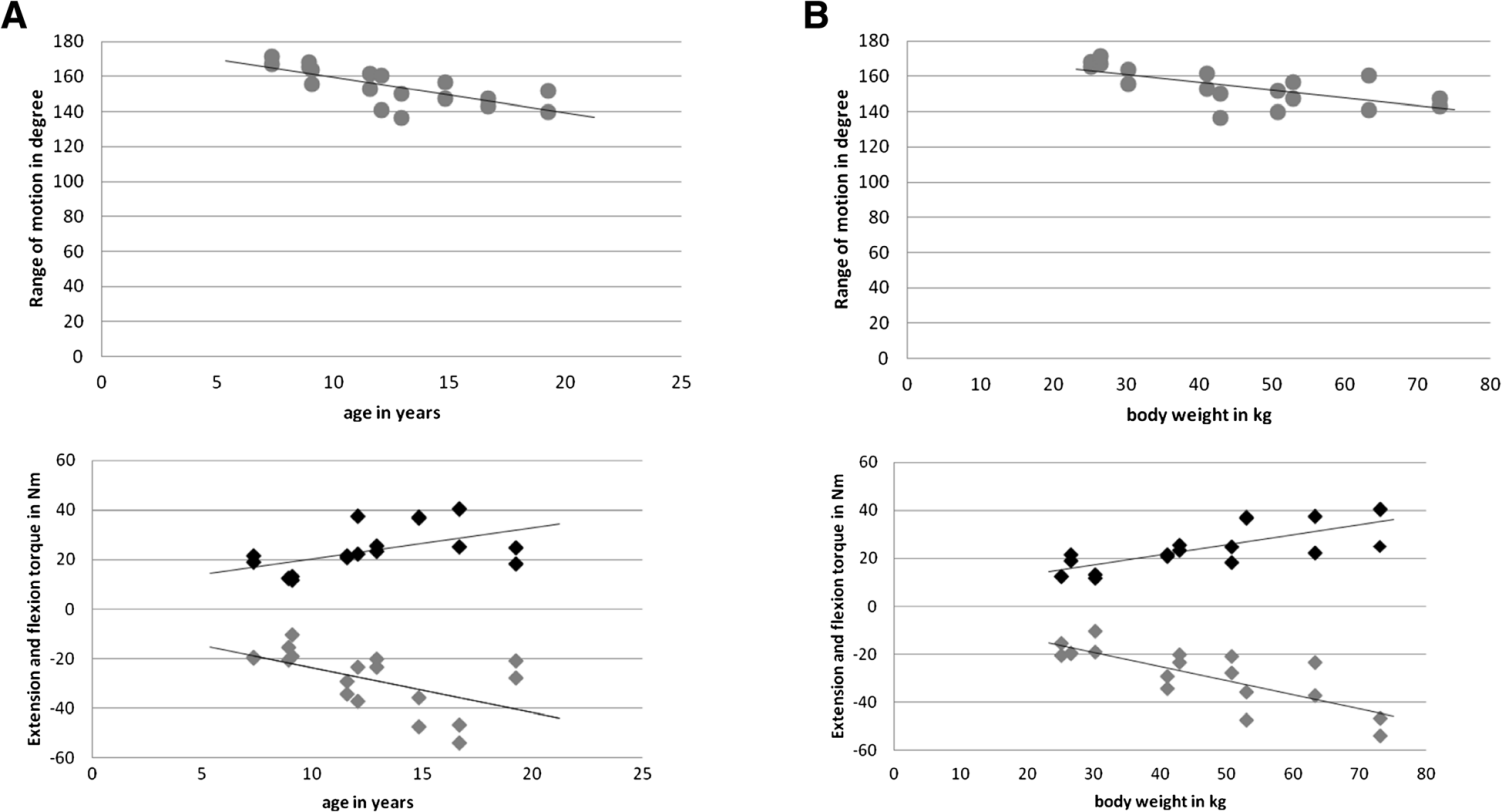
**Dynamic joint function including range of motion (ROM) and flexion and extension torque from the control cohort: Impact of age and weight. A**: Association of dynamic joint function and age in healthy controls. Dynamic joint function, including ROM, flexion and extension torques correlated with age in the healthy controls. Overall, the ROM decreased with increasing age, while both flexion and extension torque increased. **B**: Association of dynamic joint function and body weight in healthy controls. Dynamic joint function, including ROM, flexion and extension torques correlated with body weight in the healthy controls. ROM decreased with higher body weight, while both flexion and extension torque increased.

- Gender: There was no significant influence of gender on dynamic joint function. Overall, the ROM for healthy boys was slightly lower at 152.4° (range 136.6° to 163.6°) compared to 162.8° for healthy girls (140.9° to 171.2°). The median flexion torque in males was 22.6 Nm (11.7 Nm to 37.2 Nm) and 21.9 Nm (12.4 Nm to 40.3 Nm) in females; the median extension torque −25.75 Nm (−10.5 Nm to −47.5 Nm) in males and −22 Nm (−15.6 Nm to −53.9 Nm) in females.
- Age: Dynamic joint function and age were clearly correlated: With increasing age the ROM decreased, while both flexion and extension torques increased in healthy children (Figure 2A).
- Weight: Dynamic joint function and bodyweight were closely correlated: The ROM decreased with rising bodyweight. In contrast, both flexion and extension torque increased significantly with increasing body weight (Figure 2B).
- Handedness: There was no significant impact of handedness on dynamic joint function with slightly higher ROM and torque in the dominant knee.

### Dynamic joint function in children with JIA

**Figure 3:**
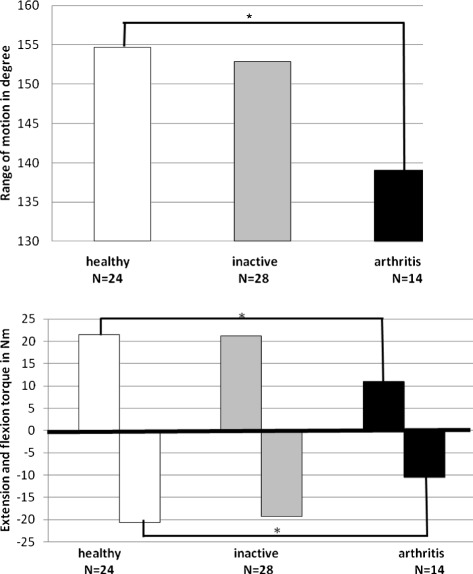
**Dynamic joint function assessments of knees with active or inactive arthritis and unaffected, healthy joints.** Knee joints with active arthritis had a significantly decreased dynamic joint function compared to healthy joints (p < 0.05). Joints with inactive arthritis demonstrated close to normal ROM, flexion and extension torques. Significant differences were indicated by asterisks.

- ROM: Children with active knee arthritis had a significantly decreased dynamic joint function compared to healthy (p < 0.05). The median ROM in active knee arthritis was only 139.1°, range 108.9° to 168.7°. This was significantly lower than unaffected joints with a median of 154.7°, range 139.7° to 168.8° and reduced when compared to inactive joints with a median of 152.9°, range 122.8° to 175.3° (Figure 3).
- Torques: Children with active arthritis had a significantly lower flexion and extension torque than healthy (p < 0.05). The median flexion torque in active knee arthritis was only 11.1 Nm (range 7.9 Nm – 34.0 Nm), compared to 21.5 Nm (range 7.4 Nm – 55.7 Nm) in unaffected joints and 21.2 Nm (range 8.7 Nm – 52.5 Nm) in inactive arthritis. Similarly, the extension torque was significantly lower in knees with active arthritis at only −10.5 Nm (range −3.3 Nm to −40.8 Nm), compared to −20.6 Nm (range −6.6 Nm to −53.9 Nm) in unaffected joints and −19.3 Nm (range −8.4 Nm to −98.6 Nm) in inactive arthritis (Figure 3).

### Within-JIA-patient comparison of the dynamic joint function

**Figure 4:**
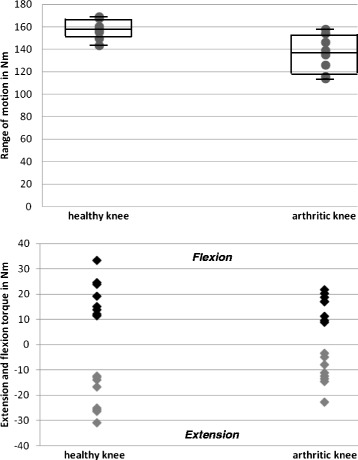
**Within-patient comparison of dynamic joint function of healthy and arthritic knees including range of motion, flexion, and extension torque in children with JIA.** Within-patient comparison of dynamic joint function of healthy and arthritic knees including range of motion, flexion, and extension torque in children with JIA. Intra-individual comparison of ROM, flexion and extension torque of healthy and arthritic knees in children with JIA reveals significant differences in all parameters of dynamic joint function. Boxes represent the interquartile range (25-75th percentile), lines within the boxes the medians. Bottom and top bars indicate the 10th and 90th percentile, respectively.

- ROM: In the inception cohort the majority of patients (7/8) with active arthritis had a significantly reduced ROM in the affected knee joint compared to the healthy knee (p < 0.05). The median difference was 20.4° (range −0.8 - 52.8°). Only one child had a similar ROM in both knees (157.2°, 158.0°), which was within the measurement error of 2°. Overall, ROM measurements of arthritic knee joints were spread wider as compared to healthy joints (Figure 4A).
- Flexion and extension torques: All but one child had a mildly decreased flexion torque in the affected joint compared to the contralateral knee. The medium flexion torque in the arthritic knee was 17.1 Nm (range 8.8 Nm - 21.7 Nm) compared to 17.2 Nm in the healthy knee joint (range 11.5 Nm - 33.4 Nm). In contrast, the extension torque was significantly reduced in the affected knee in all patients with a medium torque of −11.8 Nm (range −3.3 Nm to −22.8 Nm) compared to the median in the healthy knee joint of −20.9 Nm (range −12.5 Nm to −30.8 Nm) (p < 0.05) (Figure 4A).

### Responsiveness to change – serial dynamic joint function measurements

Serial measurements in children with JIA at different stages of disease activity determined the responsiveness to change of the dynamic joint function assessment. Of 14 patients with arthritis at time of first measurement, five completed two follow up measurements and had achieved a state of “inactive arthritis” in the previously affected knee joint at time of third measurement.

**Figure 5:**
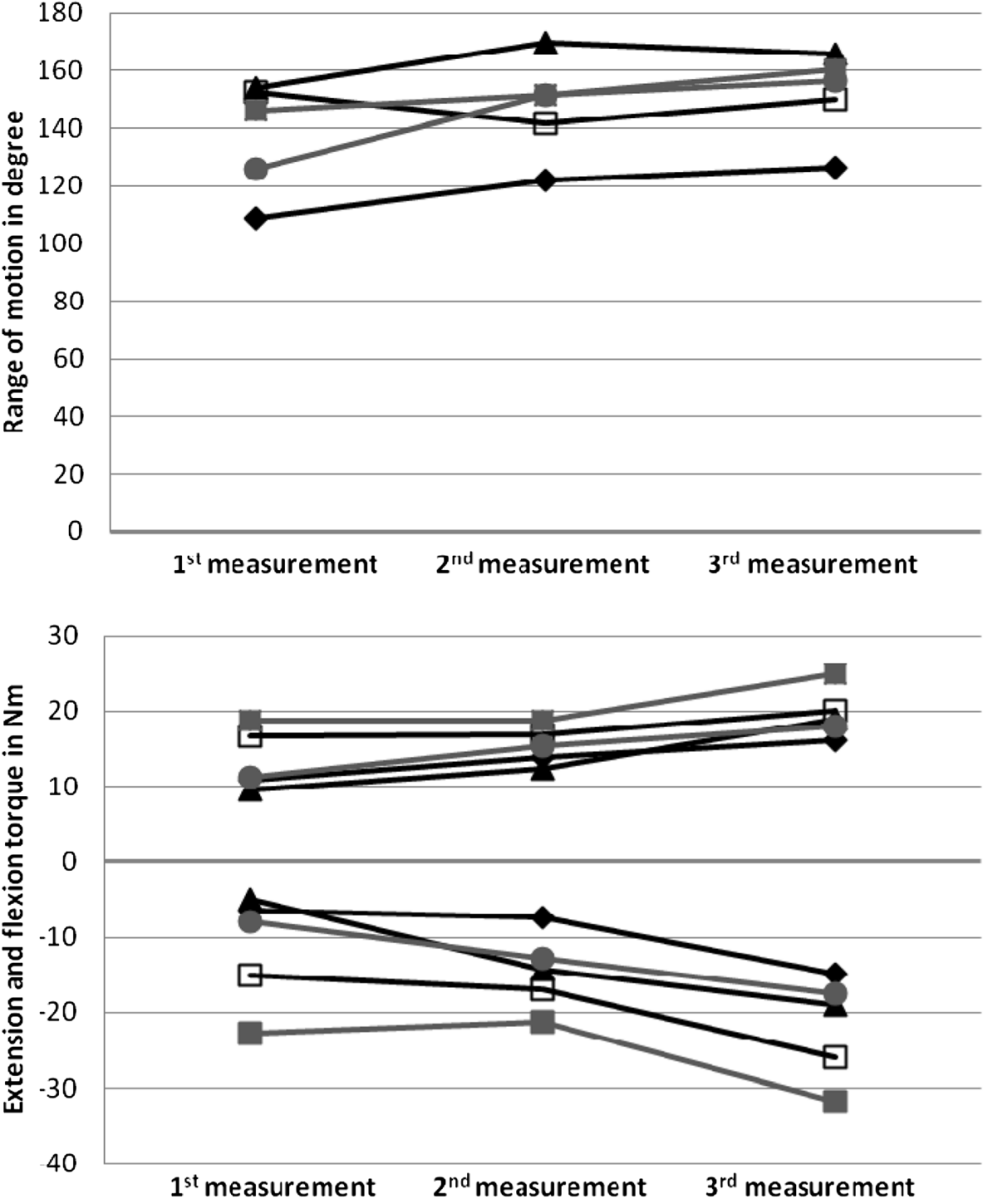
**Individual course of treatment responsiveness in children with JIA, trajectories and treatment responsiveness of dynamic joint function in JIA.** Individual course of treatment responsiveness in children with JIA, trajectories and treatment responsiveness of dynamic joint function in JIA. Individual treatment response and change in dynamic joint function was documented in five JIA patients. At time of first measurement all children had active arthritis, while inactive disease was documented at time of last measurement. Graphs reveal mild improvement in all parameters of dynamic joint function.
- Torques: Both flexion and extension torque improved with decreasing disease activity in all children. Overall the improvement was more pronounced in the extension torque. The median extension torque in active arthritis was −7.9 Nm (first measurement) and improved significantly to −19.0 Nm with inactive disease (third measurement). The medium flexion torque was 11.3 Nm in knees with active disease (first measurement), it increased to 19.0 Nm with disease control (third measurement). Data are shown in Figure 5.

- ROM: With improvement of JIA disease activity in the affected joint, the ROM increased in all 5 patients with serial measurements (Figure 5). The median ROM improved from 146.0° at time of active arthritis (first measurement) to 156.3° at time of inactive disease (third measurement).

## Discussion

This is the first study to evaluate the role of dynamic knee joint function assessments in children with JIA, and healthy controls using splint fixed electrogoniometry. The study suggests that measuring important components of knee joint function including active ROM, acceleration, flexion and extension torque with electrogoniometers is feasible in children with JIA. The measurements may offer additional clinically important information: The study determined that dynamic joint function in a group of healthy children was influenced by age and body weight; gender and handedness had no significant impact. The study results established that ROM and torque were significantly lower in arthritic knee joints as compared to healthy knee joints. Dynamic joint function assessments were responsive to change in disease activity; in particular the extension torque had a high sensitivity compared to ROM and flexion torque. At this point measurement of dynamic knee joint function in children with rheumatic diseases with electrogoniometers has to be further validated focusing on intra- and interobserver reliability to determine its utility in clinical care.

Measuring dynamic joint function with electrogoniometry was a reliable technique, not confounded by the assessor. Dynamic joint function was influenced by age and body weight in the healthy joint cohort. These findings confirm evaluation of muscle function in children and adolescents using other measurement techniques such as dynamometry [30,31]. The influence of gender was limited in our cohort; as shown for grip force and one-leg-hopping in a cohort of 868 healthy children by Lang [32]. Similar to data generated in adults, the influence of handedness on ROM in the lower extremity was insignificant [33]. Muscle torque was equally unaffected by handedness in our population.

Objective measurement tools to access joint function in children with JIA are limited. In some clinical settings hand-held-goniometry is a used tool to measure ROM in daily practice [23,34]. Manual muscle testing is highly observer dependent [35]. The most commonly used instrument for objective muscle testing is the hand-held dynamometer. However, this tool can only measure the maximal isometric grip force. The accuracy depends on the angle of the forearm during measurement and the children’s age [30,31]. Isokinetic muscle strength measurement is a technique to quantify strength in a constant velocity, which is not used in daily life activities. The measurement is complex, time-consuming and requires the correct seating of the measured person inclusive strapping to the seat [36], a procedure frightening for the children. Measurement of joint function in clinical routine often consists of a visual estimate of passive ROM and gait, which is prone to confounding, variability and inaccuracy [37].

In this study a lower dynamic joint function especially extension torque in arthritic knee joints compared to healthy was found. McKay demonstrated reduced knee extensor strength using isokinetic dynamometer in children with JIA and active arthritis [38]. Hedengren found lower joint torque values in a subgroup of children with arthritis [19]. Giannini demonstrated a significantly lower peak isometric knee extensor torque in children with arthritis using dynamometry [39]. Roth showed that the muscle force as well as the muscle mass is reduced in children with JIA [40]. This study demonstrated that the method of dynamic joint function with electrogoniometry is a robust and feasible tool to objective joint function in clinical routine.

The vast majority of JIA joint assessments are static measurements of the ROM. However, the study confirmed that ROM was the least sensitive in indicating disease activity in JIA. The extension torque measured by dynamic joint function shown in this study was significantly reduced in knees with arthritis compared to healthy knee joints. The extension torque was a sensitive marker of knee joint arthritis compared to ROM. The importance of dynamic and investigator-independent assessment is increasingly recognized. Brostrom measured dynamic force parameters during gait analysis and found a reduction in the mean gait velocity as well as in the peak vertical forces during heel contact and push-off in children with JIA compared to healthy controls [41]. Patients with polyarticular JIA had reduced walking speed and step length with hyperflexion in hip and knee joints as well as a reduced knee extension in 3D gait analysis [22]. The authors hypothesized that limitations in movement might be improved by intensified treatment and particularly sports therapy. Three dimensional gait analyses is a possibility to quantify gait and movement restriction in a simulated setting [22]. However, it is a reliable and sensitive, but very complex, time-consuming and expensive tool to measure kinetic and kinematic data of walking. In our study population a rise of ROM and flexion and extension torque from healthy joints to arthritic joints was found. Joints in remission still slightly had a diminished joint function in comparison to healthy joints. Our data show that extension torque is a much more sensitive marker for joint disease activity than ROM.

There were limitations of this study. The study cohort was small; however this represents the largest clinic-based cohort of JIA patients studied for their dynamic joint function. The duration of illness was variable in our group. The dynamic joint function results may therefore have represented both residual active arthritis and possibly early damaged joints. No corresponding MRI imaging studies were conducted.

## Conclusions

In conclusion, the study demonstrated that the electrogoniometric measurement of dynamic flexion and extension torque can be easily performed and is an excellent method to demonstrate a difference between healthy and arthritic knee joints. Reduction in ROM and flexion and extension torque was found early in disease. Electrogoniometric measurements are able to estimate the ROM objectively. Dynamic flexion and extension torque can be measured using electrogoniometry in an easy to handle setting and in an open-end of joint positions as well as during any action. Based on the encouraging results of this descriptive study, the validity of the new method should be further tested including intra and inter-rate reliability testing and formal comparative studies with other methods of joint assessemnts and muscle strength evaluation.

### Consent

Written informed consent was obtained from the patient’s parent or guardian for the publication of this report and any accompanying images.
